# Giant adenomatoid odontogenic tumor of maxilla—a case report exploring the radiological imaging aspects and pathway to diagnosis with review of literature

**DOI:** 10.1093/bjrcr/uaag027

**Published:** 2026-07-08

**Authors:** Sakshi Agrawal, Abhinav Chander Bhagat, Anshul Rai, Ujjawal Khurana, Raj Barfa, Radha Sarawagi Gupta, Rajesh Malik

**Affiliations:** Department of Radiodiagnosis, All India Institute of Medical Sciences, Bhopal 462020, India; Department of Radiodiagnosis, All India Institute of Medical Sciences, Bhopal 462020, India; Department of Dentistry, All India Institute of Medical Sciences, Bhopal 462020, India; Department of Pathology and Lab Medicine, All India Institute of Medical Sciences, Bhopal 462020, India; Department of Radiodiagnosis, All India Institute of Medical Sciences, Bhopal 462020, India; Department of Radiodiagnosis, All India Institute of Medical Sciences, Bhopal 462020, India; Department of Radiodiagnosis, All India Institute of Medical Sciences, Bhopal 462020, India

**Keywords:** adenomatoid odontogenic tumor, giant, two-third tumor, maxilla, calcification, canine, dentigerous cyst, calcifying odontogenic cyst, calcifying epithelial odontogenic tumor

## Abstract

Adenomatoid odontogenic tumor is a rare benign odontogenic neoplasm, comprising around 3% of all odontogenic tumors and <1% of all jaw tumors. It affects predominantly adolescents and young adults, with a remarkable female preponderance. This tumor most commonly tends to arise from the anterior part of maxilla and is frequently seen in association with an unerupted canine tooth, clinically mimicking a dentigerous cyst. This case report describes a giant adenomatoid odontogenic tumor of the maxilla highlighting the CT and MR imaging characteristics of this rare entity and explores the differential diagnoses with key differentiating features along with a brief review of literature.

## Introduction

Adenomatoid odontogenic tumor (AOT) is a rare benign odontogenic neoplasm characterized by distinct clinical and radiological features. Arising from the odontogenic epithelium, it represents approximately 3% of all odontogenic tumors and predominantly affects adolescents and young adults, with a marked predilection for females.^1^ This tumor most frequently arises in the anterior regions of the maxilla and mandible and is often associated with an unerupted tooth, which is usually a canine, clinically mimicking a dentigerous cyst.^2^ In this case report, we revisit this rare entity along with a brief review of literature and describe an unusually large adenomatoid odontogenic tumor focusing on the imaging characteristics on computed tomography (CT) and magnetic resonance (MR) imaging, explore the differential diagnoses with pertinent differentiating points, and elucidate the implications on management.

## Case report

### Clinical history

**Figure 1:**
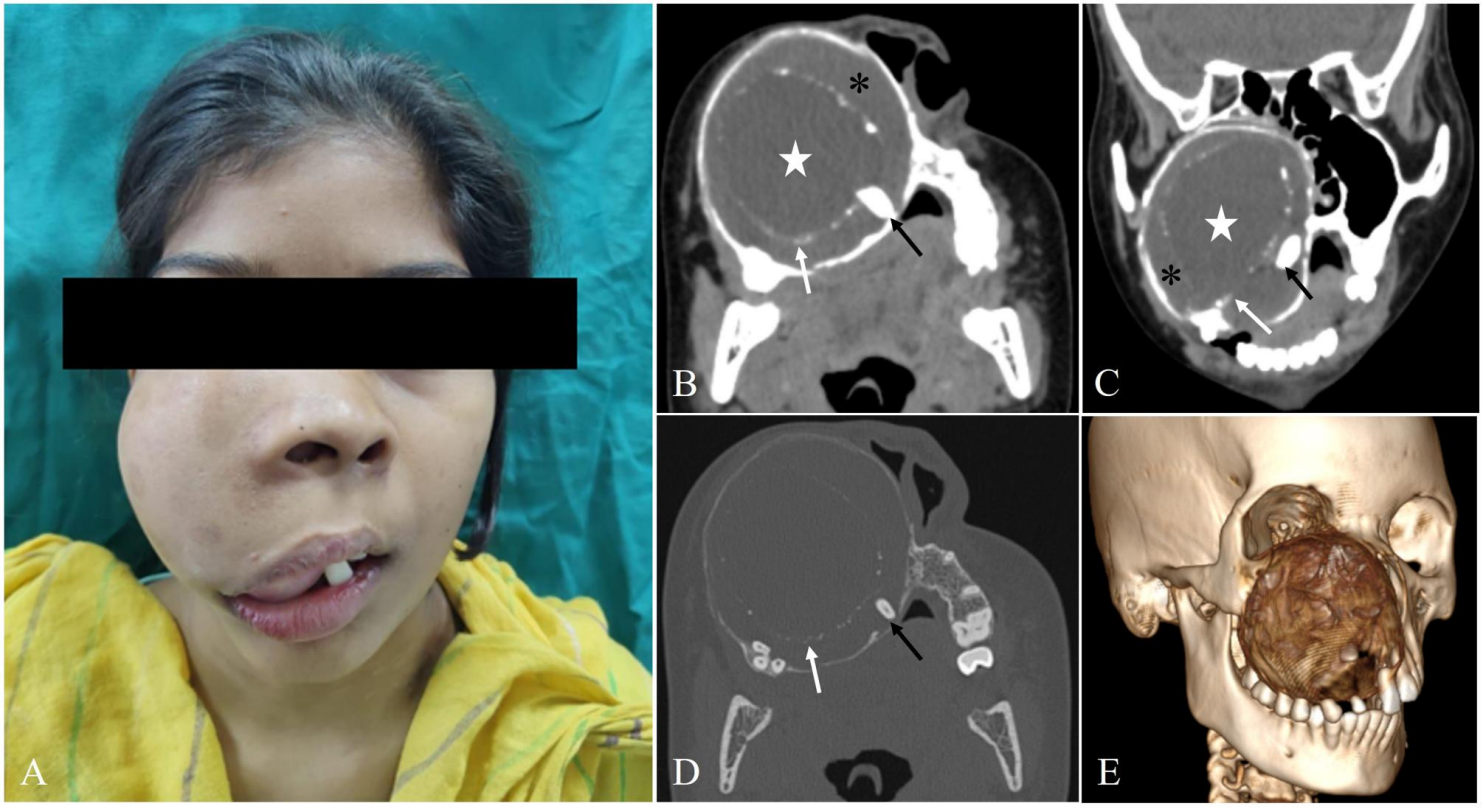
(A-E) Clinical photograph (A) shows a large swelling in the right maxillary region. Non-contrast axial (B) and coronal (C) CT in soft tissue window and axial bone window (D) reveals a well-defined, unilocular, intra-osseous expansile lesion arising from the anterior aspect of right maxilla. The lesion shows hypodense cystic component centrally (star). Peripheral rim of discrete calcific foci (white arrows) is seen, which is further surrounded by a soft-tissue density capsule (asterisk). The crown and root of the unerupted canine (black arrows) is completely engulfed by the lesion. 3-D VRT (E) image shows the lesion involving the right maxilla.

An adolescent female patient presented with complaints of progressively increasing painless swelling in the right maxillary and nasal region for 2 years, with a recent onset of pain in the last 1 month. She was afebrile and had no history of any previous tooth extraction or trauma, or any dental infection. Extra-oral examination revealed right-sided maxillary swelling, causing an elevation of the nostrils and resultant cosmetic disfiguration (Figure 1). Upon palpation, the swelling was hard in consistency, non-compressible, non-tender, and immobile. On intraoral examination, right-sided maxillary teeth were irregularly aligned and mal-occluded due to the mass effect of the lesion.

### Radiological evaluation

The patient was referred for imaging and underwent a non-contrast CT examination. CT revealed a large, oval-shaped, expansile, unilocular hypodense cystic (mean attenuation 17 HU) lesion measuring 7 × 7 × 6 cm arising from the anterior half of right maxillary alveolar arch and completely enveloping the right canine (Figure 1). The lesion was projecting into and obliterating the right maxillary sinus. A rim of tiny specks of calcification was seen at the periphery of the cystic component which was further surrounded by a circumferential capsule of soft-tissue (mean attenuation 31 HU) density. The lesion was causing smooth osseous expansion, cortical thinning, and bony remodeling of the maxilla, with few focal areas of bony dehiscence. However, no evidence of any aggressive bony destruction, erosion, periosteal reaction or infiltration into the adjacent structures was noted. There was displacement of the underlying right-sided maxillary teeth, and the lesion was protruding into the right half of the oral cavity infero-medially. Root resorption of the right-sided maxillary central and lateral incisors was also noted. A provisional diagnosis of odontogenic tumor was made, and in view of the recent onset perception of pain, possibility of malignant degeneration was suspected; hence, the patient was further evaluated with a contrast-enhanced MR imaging of the face.

**Figure 2:**
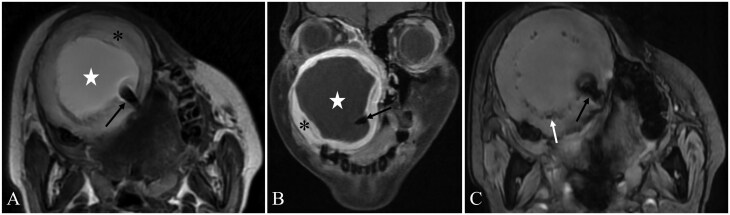
(A-C) Axial T2W (A) MR image reveals central hyperintense cystic component (star) surrounded by a peripheral thick rind of intermediate to mildly hyperintense soft tissue (asterisk). Coronal (B) post-contrast T1W fat-suppressed image shows homogeneous enhancement of the soft tissue capsule (asterisk) with non-enhancing central cystic region (star). Axial GRE (C) image shows punctate foci of blooming (white arrow) surrounding the cystic component, corresponding to calcification. Engulfed unerupted canine (black arrows) is seen along the infero-medial aspect of the lesion.

MR imaging was performed on a 3 T scanner using a head coil. T1-weighted (T1W) spin-echo and T2-weighted (T2W) fast-spin echo with and without fat suppression technique, diffusion-weighted imaging (DWI), gradient-recalled echo (GRE), and pre-and post-contrast T1 sequences (with fat-suppression) were obtained after intravenous administration of 0.2 mL/kg (0.1 mmol/kg) gadodiamide. The lesion revealed central cystic component, showing T1W intermediate to hypointense signal and T2W hyperintense signal without diffusion restriction on DWI. A thin peripheral rim of blooming was seen on the GRE sequence which was suggestive of calcification (Figure 2). Surrounding it circumferentially was a thick rind of soft tissue showing intermediate signal on T1W images and intermediate to mildly hyperintense signal on T2W images. This soft-tissue capsule measured 1.6 cm in maximum thickness and showed homogeneous post-contrast enhancement.

Based on the CT and MR imaging findings, the relevant differential diagnoses considered were calcifying odontogenic cyst (COC, or Gorlin cyst), calcifying epithelial odontogenic tumor (CEOT, or Pindborg tumor), and AOT (Table 1). In view of the typical location, patient demographics, and imaging features, AOT was suggested as the most likely diagnosis.

**Table 1:** Differentiation of adenomatoid odontogenic tumor from calcifying odontogenic cyst and calcifying epithelial odontogenic tumor.

Feature	Adenomatoid odontogenic tumor	Calcifying odontogenic cyst	Calcifying epithelial odontogenic tumor
**Demographics**	2^nd^-3^rd^ decades, predominantly females	2^nd^-3^rd^ decades, no gender predilection	4^th^-6^th^ decades, no gender predilection
**Typical location**	Anterior maxilla (canine region), up to a third seen in mandible	Almost equally in the maxilla (strong predilection for anterior aspect in canine region) and mandible	Body of mandible (premolar and molar region), up to a third seen in maxilla
** CT appearance**	Well-defined hypodense lesion with peripheral rim of calcification	Well-circumscribed hypodense lesion with peripheral foci of calcification, often associated with odontomas	Circumscribed or poorly marginated lesion with scattered calcifications
**Calcification pattern**	Scattered or rim of speckled, punctate calcifications	Irregular, mass-like calcification towards one side of the lesion	“Driven snow” pattern of calcification representing radiating cluster of calcific densities
**Histopathology**	Lesion with fibrous capsule and central nodules showing formation of duct-like structures with calcification and amyloid	Lesion with cystic architecture and ghost cells which often calcify	Sheets, islands and cords of polyhedral cells with distinct cell borders, islands of odontogenic epithelium with focal calcification and amyloid
**Classification as per WHO 5th edition (2022)**	Benign epithelial odontogenic tumor	Odontogenic cyst	Benign epithelial odontogenic tumor

### Surgical intervention and histopathological confirmation

**Figure 3:**
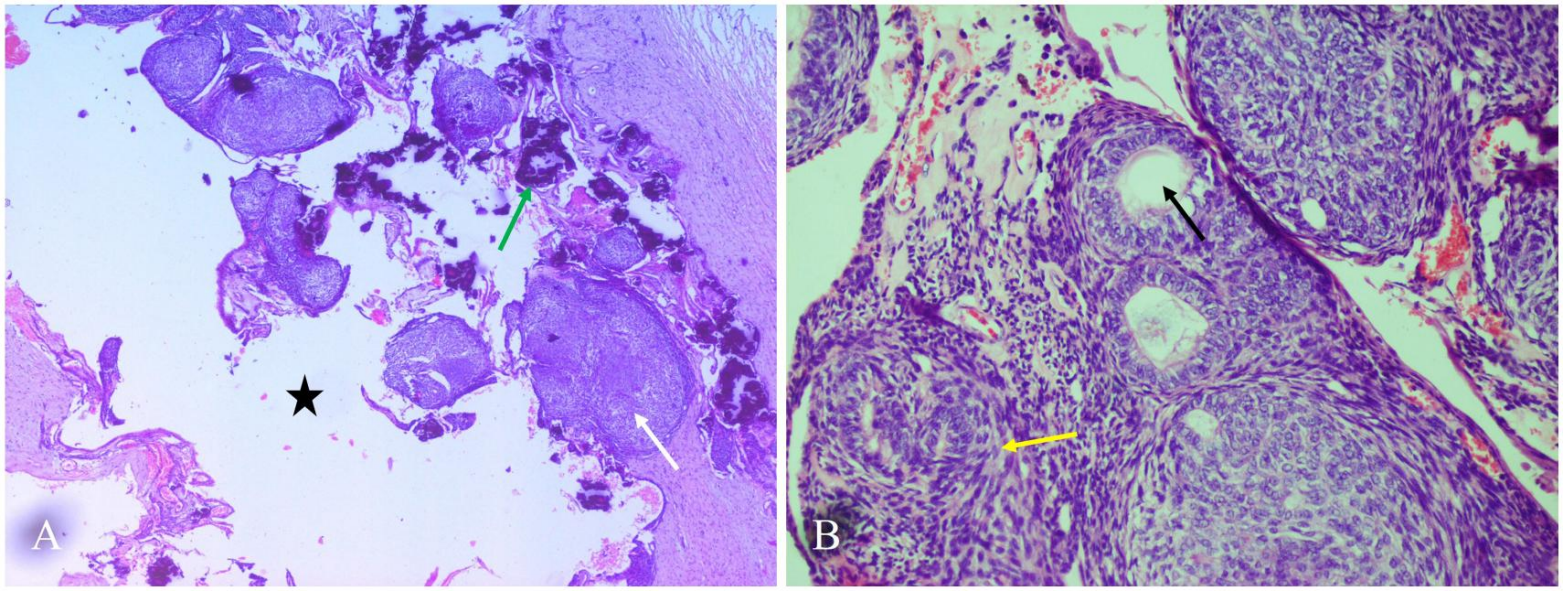
(A, B) Photomicrographs of post-surgical specimen show cyst-like structure formation (star) and cystic mural nodules (white arrow). The mural nodule is composed of epithelial cells forming duct-like structures (black arrow) and whorls (yellow arrow). Also seen are calcifications (green arrow), leading to sectioning artifacts. The wall shows fibrous capsule.

**Figure 4:**
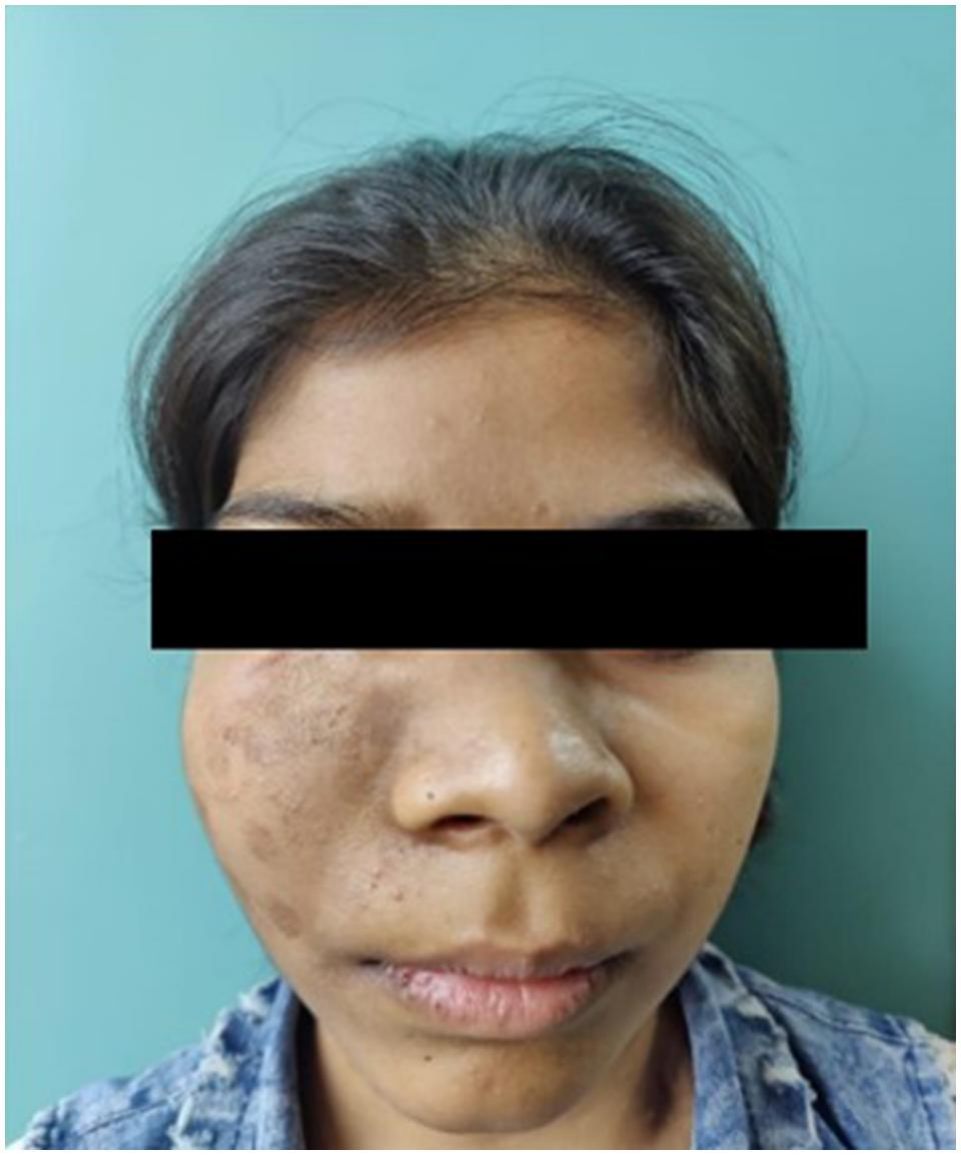
Post-operative 6-month follow-up photograph of the patient.

The patient underwent enucleation of the lesion, and the diagnosis of AOT was confirmed on histopathological examination. It revealed cyst-like structure formation and cystic mural nodules composed of epithelial cells forming duct-like structures, cribriform patterns and whorls, together with benign fibroblast proliferation along with calcifications and peripheral young bone formation with osteoblastic rimming (Figure 3). The patient is currently under follow-up and is doing well with no evidence of recurrence 6 months post-surgery (Figure 4).

## Discussion

### Terminology and histopathological classification

Adenomatoid odontogenic cysts are an interesting hamartomatous malformations, having been described using a variety of terminologies over the years. Various authors have made different attempts to designate this entity. At first, Steensland in 1905 described it as “epithelioma adamantium.”^3^ Unal et al.^4^ described it under various names—adenoameloblastoma, cystic complex composite odontoma, ameloblastic odontogenic tumor, and odontogenic adenomatoid tumor.

The term “Adenomatoid odontogenic tumor”, first coined by Philipsen and Birn^5^ in 1969, is the most commonly used. World Health Organization (WHO) adopted this terminology in 1971 and defined it as “A tumor of odontogenic epithelium with duct-like structures and with varying degrees of inductive changes in the connective tissue. The tumor may be partly cystic, and in some cases, the solid lesion may be present only as masses in the wall of a large cyst. It is generally believed that the lesion is not a neoplasm.”^6^ Odontogenic tumors can develop from epithelium and/or ectomesenchyme which give rise to the tooth-forming apparatus.^7^ According to the latest 2022 WHO classification (5^th^ edition) of odontogenic lesions, AOT is considered as a tumor under the group of benign epithelial odontogenic tumors.^8^

### Demographics and clinical features

AOT is an atypical tumor of odontogenic origin that comprises about 0.1% of jaw tumors and accounts for 2%-7% of all odontogenic tumors. It is more common in females (F: M = 2.3:1) and frequently presents in the second decade of life. It has a predilection for anterior maxilla (maxilla: mandible = 2.6:1) and is often seen in association with an unerupted tooth.^9^ This most commonly is a canine; however, more than 1 tooth may also be involved. Few cases of mandibular location of AOT have also been described.^1^^,^^10–12^ Most lesions vary in size between 1 and 3 cm, although they can present with much larger dimensions owing to higher growth rate in the younger age-group and delay in seeking medical attention.^13^^,^^14^

Clinically, the patients present with slow-growing, painless, and hard swelling of the face causing displacement of the teeth, leading to it being frequently misdiagnosed as an odontogenic cyst. Although it is classically described as not causing root resorption of adjacent teeth, larger lesions may do so.^12^ AOT affects young females in two-third cases, around two-third cases develop in the maxilla, two-third cases are related to an unerupted tooth, canines are affected in about two-third cases and two-third cases show calcifications; earning them the moniker of “two-third tumor.”^3^

### Clinico-pathological variants

On the basis of location and growth pattern, 3 variants of AOT are described: follicular, extrafollicular, and peripheral. The follicular (pericoronal) variety is the most common, seen in approximately 70% of cases, presenting as an intraosseous lesion in association with an unerupted tooth. The extrafollicular (extracoronal) variant, comprising 25% of cases, also presents as an intraosseous lesion; however, it is not associated with an unerupted tooth. The peripheral (extra-osseous/gingival) variety arises in the gingival tissue and is rare, representing 5% of all AOTs.^15^ This sub-type is not easily detected radiographically and may appear as scalloping or indentation on the margin of the underlying bone.

Our case followed the trend and was of follicular subtype, associated with an unerupted canine.

### Clinico-radiological features and differential diagnoses

AOTs and dentigerous cysts are frequently confused due to their overlapping epidemiological and locational characteristics, as well as some shared imaging features. Both lesions predominantly affect adolescents and young adults, often presenting in the anterior regions of the maxilla and mandible, which is a common site for both conditions.^16^ This demographic overlap and similar anatomical predilection can lead to initial misdiagnosis when evaluating radiographic images.

On imaging, both AOTs and dentigerous cysts typically appear as well-defined radiolucent lesions associated with the crown of an unerupted tooth, which further complicates differentiation (Table 2). The fine calcifications within the AOT may not be visualized on standard radiographs, however intraoral periapical radiograph can show calcifications as discrete radio-opacities with a flocculent pattern within the radiolucency even with minimal calcified deposits, which are present in approximately 78% of the lesions.^1^^,^^17^ Also, an AOT envelops both the crown and the root of the tooth, whereas a dentigerous cyst surrounds only the crown.^18^ Understanding these subtleties is crucial for accurate diagnosis. Crucially, AOT is distinguished by its characteristic peripheral rim of calcification and internal punctate or speckled calcifications on CT, central cystic component showing intermediate to low signal on T1W MR imaging with high signal on T2W MR imaging and homogeneous post-contrast enhancement of peripheral circumferential soft-tissue capsule.

**Table 2:** Differentiation of adenomatoid odontogenic tumor from dentigerous cyst.

Feature	Adenomatoid odontogenic tumor	Dentigerous cyst
**Demographics**	Adolescents and young adults, predominantly females	Adolescents and young adults, slightly higher incidence in males
**Typical location**	Anterior maxilla, canine (pericoronal), up to a third seen in mandible	Mandible, third molar (pericoronal), up to a third seen in maxilla (canine region)
**CT appearance**	Well-defined hypodense lesion with peripheral rim of calcification	Well defined hypodense lesion surrounding the crown of an unerupted tooth
**Calcification pattern**	Scattered or rim of speckled, punctate calcifications	Calcification typically absent
**MR imaging appearance**	Central cystic component with intermediate to low signal intensity on T1W images and high signal intensity on T2W images Peripheral thick rind of soft tissue surrounding the calcification showing intermediate signal intensity on T2W images and homogeneous post-contrast enhancement	Low signal intensity on T1W images and high signal intensity on T2W images, reflective of completely cystic nature with no soft tissue component Usually, no significant enhancement of the cyst wall on post-contrast images
**Histopathology**	Lesion with fibrous capsule and central nodules showing formation of duct-like structures with calcification and amyloid	Cyst lined with non-keratinized stratified squamous epithelium, having a thin fibrous wall with or without inflammation
**Classification as per WHO 5th edition (2022)**	Benign epithelial odontogenic tumor	Odontogenic cyst

COC, a developmental odontogenic cyst, also shows internal calcifications. However, the pattern of calcification is different in COC, appearing as irregular and mass-like towards one side of the lesion, often associated with odontomas. In contrast, AOT usually demonstrates a fine peripheral rim of calcification arranged in a circular fashion. Furthermore, a radiolucent band representing the soft tissue capsule is seen peripheral to the calcification in AOT.^19^

Well-defined corticated margins help differentiate AOT from other odontogenic tumors such as CEOT and ameloblastoma, which may present with less defined or more irregular radiographic features. Calcifications are rare and unusual in ameloblastoma, whereas CEOT may show the characteristic “driven snow” pattern of calcification representing radiating cluster of calcific densities arising from the central radiodense lesion.^20^^,^^21^

In summary, both CT and MR imaging play an important role in the diagnosis of AOT. CT is exceptional in detecting the calcifications while MR imaging complements CT by providing detailed information about the tumor’s internal morphology, soft tissue components, and enhancement patterns. Our case was unusual in view of exceptionally large size of the lesion and presence of root resorption of adjacent teeth. However, careful examination of the CT and MR imaging features provided the clue to correct diagnosis.

### Management strategy

AOTs are managed surgically by enucleation and curettage. Due to their generally benign and noninvasive behavior, conservative surgery suffices in the majority of cases with excellent prognosis. However, in larger lesions, partial resection of the maxilla or mandible may be required, and close follow-up is important since recurrence may occur in rare cases.^22^

## Conclusion

This case report illustrates the classic imaging features of an adenomatoid odontogenic tumor, specifically focusing on the patterns of calcification observed on CT together with morphological features and signal characteristics on MR imaging. The peripheral rim of fine speckled calcification surrounded by a circumferential rind of enhancing soft tissue are key imaging features that aid in diagnosing AOT. Location within the anterior maxilla and associated unerupted tooth within the lesion, usually a canine, is also very characteristic. Unusual features like large size and root resorption of adjacent teeth may also be seen occasionally, as in this case. CT and MR imaging findings are not only crucial for accurate diagnosis but also for planning effective treatment strategies. Even though it is a rare entity, understanding these features enhances the radiologist’s ability to differentiate AOT from other odontogenic lesions and ensures optimal patient management.

## Learning points

- Adenomatoid odontogenic tumor is a rare benign odontogenic neoplasm, which can be misdiagnosed clinically as a dentigerous cyst.
- Although unusual, the lesion may grow to an exceptionally large size and cause root resorption of adjacent teeth.
- Key CT and MR imaging features include peripheral rim of fine speckled calcification surrounded by a circumferential rind of enhancing soft tissue with a central cystic component, helping in differentiation from lesions like calcifying odontogenic cyst and calcifying epithelial odontogenic tumor.
- It is managed surgically by enucleation and curettage; however larger lesions may necessitate partial resection of the maxilla or mandible.

## References

[1] More CB , Das S, Gupta S, Bhavsar K. Mandibular adenomatoid odontogenic tumor: radiographic and pathologic correlation. J Nat Sci Biol Med. 2013;4:457-462. 10.4103/0976-9668.116965 24082751 PMC3783799

[2] Dayi E , Gürbüz G, Bilge OM, Ciftcioğlu MA. Adenomatoid odontogenic tumour (adenoameloblastoma). Case report and review of the literature. Aust Dent J. 1997;42:315-318. 10.1111/j.1834-7819.1997.tb00136.x 9409047

[3] Philipsen HP , Reichart PA. Adenomatoid odontogenic tumour: facts and figures. Oral Oncol. 1999;35:125-131. 10.1016/s1368-8375(98)00111-0 10435145

[4] Unal T , Cetingul E, Gunbay T. Peripheral adenomatoid odontogenic tumor: birth of a term. J Clin Pediatr Dent. 1995;19:139-142. 7577734

[5] Philipsen HP , Birn H. The adenomatoid odontogenic tumour. Ameloblastic adenomatoid tumour or adeno-ameloblastoma. Acta Pathol Microbiol Scand. 1969;75:375-398. 5801660

[6] Kramer IR , Pindborg JJ, Shear M. WHO International Histological Classification of Tumors. No. 5. Histological Typing of Odontogenic Tumors, Jaw Cysts, and Allied Lesions. World Health Organization; 1971:41-42.

[7] Vijayvergiya G , Tandon A, Rai A, et al Histopathologic spectrum and clinical correlation of lesions of jaw - a series of 60 cases. Int J Clin Exp Pathol. 2022;15:467-475. 36628072 PMC9827225

[8] Soluk-Tekkesin M , Wright JM. The world health organization classification of odontogenic lesions: a summary of the changes of the 2022 (5th) edition. The world health organization classification of odontogenic lesions: a summary of the changes of the 2022 (5th) edition. Turk Patoloji Derg. 2022;38:168-184. 10.5146/tjpath.2022.01573 35578902 PMC9999699

[9] Kamble A , Shimpi MR, Dash JK, Sahoo PK, Chaudhary S, Doiphode M. Adenomatoid odontogenic tumor of the maxilla in a 13-year-old patient: a rare case report with a review of literature. Int J Clin Pediatr Dent. 2021;14:596-600. 10.5005/jp-journals-10005-1771 34824522 PMC8585894

[10] Narayanan VS , Naidu G, Ragavendra R, Mhaske-Jedhe S, Haldar M. Adenomatoid odontogenic tumor of the mandible with unusual radiographic features: a case report. Imaging Sci Dent. 2013;43:111-115. 10.5624/isd.2013.43.2.111 23807935 PMC3691371

[11] Duc NQ , Lam VN, Tien NP, Hanh NTM, Dang VDH. A giant adenomatoid odontogenic tumor of the mandible: a case report and literature review. Int J Surg Case Rep. 2022;96:107295. 10.1016/j.ijscr.2022.107295 35714392 PMC9204738

[12] Fujita A , Ueyama Y, Nagatsuka H, Kawamata H. A case of large adenomatoid odontogenic tumor in the posterior region of the mandible showing root resorption. J Oral Med Oral Surg. 2021;27:19. 10.1051/mbcb/2020053

[13] John JB , John RR. Adenomatoid odontogenic tumor associated with dentigerous cyst in posterior maxilla: a case report and review of literature. J Oral Maxillofac Pathol. 2010;14:59-62. 10.4103/0973-029X.72502 21731264 PMC3125061

[14] Mohamed A , Singh AS, Raubenheimer EJ, Bouckaert MM. Adenomatoid odontogenic tumour: review of the literature and an analysis of 33 cases from South Africa. Int J Oral Maxillofac Surg. 2010;39:843-846. 10.1016/j.ijom.2010.06.014 20638244

[15] Baskaran P , Misra S, Kumar MS, Mithra R. Adenomatoid odontogenic tumor - a report of two cases with histopathology correlation. J Clin Imaging Sci. 2011;1:64. 10.4103/2156-7514.92186 22347682 PMC3279693

[16] Takahashi K , Yoshino T, Hashimoto S. Unusually large cystic adenomatoid odontogenic tumour of the maxilla: case report. Int J Oral Maxillofac Surg. 2001;30:173-175. 10.1054/ijom.2000.0003 11405456

[17] Dare A , Yamaguchi A, Yoshiki S, Okano T. Limitation of panoramic radiography in diagnosing adenomatoid odontogenic tumors. Oral Surg Oral Med Oral Pathol. 1994;77:662-668. 10.1016/0030-4220(94)90331-x 8065735

[18] Rashmi G , Santosh G, Harshavardhan S, Praveen KM. Adenomatoid odontogenic tumour. Indian J Dent Adv. 2009;1:67-72.

[19] Chindasombatjaroen J , Poomsawat S, Kakimoto N, Shimamoto H. Calcifying cystic odontogenic tumor and adenomatoid odontogenic tumor: radiographic evaluation. Oral Surg Oral Med Oral Pathol Oral Radiol. 2012;114:796-803. 10.1016/j.oooo.2012.08.452 23159119

[20] Venkateswarlu M , Geetha P, Lakshmi Kavitha N. CT imaging findings of a calcifying epithelial odontogenic tumour. Br J Radiol. 2012;85:e14-e16. 10.1259/bjr/65485502 22190756 PMC3473932

[21] Misra SR , Lenka S, Sahoo SR, Mishra S. Giant pindborg tumor (calcifying epithelial odontogenic tumor): an unusual case report with radiologic-pathologic correlation. J Clin Imaging Sci. 2013;3:11. 10.4103/2156-7514.124056 PMC3906659 24516774

[22] Chuan-Xiang Z , Yan G. Adenomatoid odontogenic tumor: a report of a rare case with recurrence. J Oral Pathol Med. 2007;36:440-443. 10.1111/j.1600-0714.2007.00521.x 17617839

